# Poisoning after Injections of Corrosive Sublimate

**Published:** 1887-09

**Authors:** 


Poisoning after Injections of Corrosive Sublimate.—Fleischmann, the first assistant in the obstetrical clinic at Prague, adds another to the long list of deaths following the use of corrosive sublimate injections during and after labor. Two injections were given, one before and one after the examination of a young primipara; shortly after the second there was a discharge of bloody mucus from the vagina, followed by severe abdominal pain, a serous diarrhœa and vomiting of a quantity of bile; on the following day, labor was completed without difficulty, and as the symptoms of the day before were attributed to the absorption of a small quantity of the mercurial salt, carbolic acid injections were used during the after-labor. The mischief, however, had already been done, for, after exhibiting symptoms that pointed indubitably to mercurial poisoning, the woman died on the sixth day. The *post-mortem* examination showed spongy and swollen gums, ulcers on the tongue and pharynx, superficial ulcers in the ascending colon, and a high grade of acute inflammation of the kidneys. Dr. Fleischmann ends his communication in the *Centralblatt für Gynäkologie* with an impressive appeal to the profession to banish corrosive sublimate from obstetrical practice.—*Provincial Medical Journal.*

